# Ethyl 2-(2-hy­droxy-5-nitro­phen­yl)acetate

**DOI:** 10.1107/S1600536811000389

**Published:** 2011-01-29

**Authors:** Bing Guo, Ya-Bin Shi, Jin-Hua Yao, Jian-Ning Guan

**Affiliations:** a College of Science, Nanjing University of Technology, Xinmofan Road No. 5 Nanjing, Nanjing 210009, People’s Republic of China

## Abstract

In the crystal structure of the title compound, C_10_H_11_NO_5_, inter­molecular O—H⋯O hydrogen bonds link the mol­ecules into chains along the *b-*axis direction. Weak C—H.·O hydrogen bonds also occur.

## Related literature

For the use of the title compound as a pharmaceutical inter­mediate and for the preparation, see: Omar *et al.* (2003 ▶). For bond-length data, see: Allen *et al.* (1987 ▶).
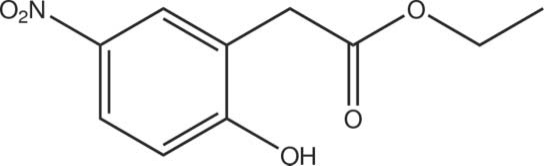


## Experimental

### 

#### Crystal data

C_10_H_11_NO_5_
*M* *_r_* = 225.20
Monoclinic, 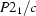

*a* = 11.066 (2) Å
*b* = 10.860 (2) Å
*c* = 8.6970 (17) Å
β = 97.85 (3)°
*V* = 1035.4 (4) Å^3^
*Z* = 4
Mo *K*α radiation
μ = 0.12 mm^−1^
*T* = 293 K
0.30 × 0.20 × 0.10 mm

#### Data collection

Enraf–Nonius CAD-4 diffractometer
Absorption correction: ψ scan (North *et al.*, 1968 ▶) *T*_min_ = 0.966, *T*_max_ = 0.988
3894 measured reflections
1905 independent reflections
1382 reflections with *I* > 2σ(*I*)
*R*_int_ = 0.056
3 standard reflections every 200 reflections  intensity decay: 1%

#### Refinement

*R*[*F* ^2^ > 2σ(*F* ^2^)] = 0.057
*wR*(*F* ^2^) = 0.153
*S* = 1.00
1905 reflections
145 parameters
H-atom parameters constrained
Δρ_max_ = 0.59 e Å^−3^
Δρ_min_ = −0.19 e Å^−3^

### 

Data collection: *CAD-4 EXPRESS* (Enraf–Nonius, 1989 ▶); cell refinement: *CAD-4 EXPRESS*; data reduction: *XCAD4* (Harms & Wocadlo, 1995 ▶); program(s) used to solve structure: *SHELXS97* (Sheldrick, 2008 ▶); program(s) used to refine structure: *SHELXL97* (Sheldrick, 2008 ▶); molecular graphics: *SHELXL97*; software used to prepare material for publication: *PLATON* (Spek, 2009 ▶).

## Supplementary Material

Crystal structure: contains datablocks global, I. DOI: 10.1107/S1600536811000389/bq2266sup1.cif

Structure factors: contains datablocks I. DOI: 10.1107/S1600536811000389/bq2266Isup2.hkl

Additional supplementary materials:  crystallographic information; 3D view; checkCIF report

## Figures and Tables

**Table 1:** Hydrogen-bond geometry (Å, °)

*D*—H⋯*A*	*D*—H	H⋯*A*	*D*⋯*A*	*D*—H⋯*A*
O3—H3*A*⋯O2^i^	0.82	1.93	2.749 (2)	180
C2—H2*A*⋯O3^ii^	0.97	2.60	3.340 (3)	134
C4—H4*A*⋯O4^iii^	0.97	2.54	3.431 (3)	153
C6—H6*A*⋯O5^iv^	0.93	2.51	3.351 (3)	151
C8—H8*A*⋯O4^v^	0.93	2.59	3.430 (3)	150

Symmetry codes: (i) 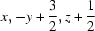; (ii) 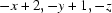; (iii) 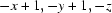; (iv) 
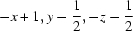
; (v) 
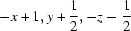
.

**Fig. 1.**
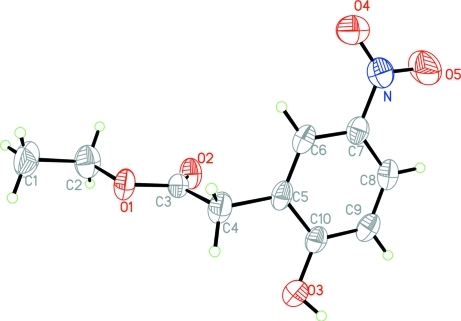
The molecular structure of the title molecule, with the atom-numbering scheme. Displacement ellipsoids are drawn at the 50% probability level.

**Fig. 2.**
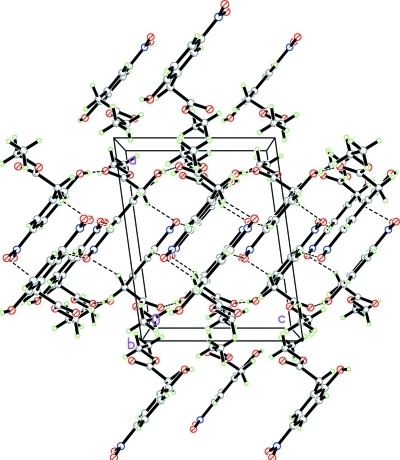
A packing diagram of (I). Hydrogen bond is shown as dashed line.

## supplementary crystallographic information

### Comment

The compound, 5-nitrobenzofuran-2(3H)-one, which is an effective intermediate prepared dronedarone, plays an important role in the fields of natural products and medicinal chemistry. The title compound, ethyl 2-(2-hydroxy-5-nitrophenyl)acetate, (I), is a useful pharmaceutical intermediate (Omar *et al.*, 2003).

In the molecule of the title compound (Fig. 1), the bond lengths (Allen *et al.*, 1987) and angles are within normal ranges. In the crystal structure, intermolecular O-H···O hydrogen bonds (Table 1.) link the molecules forming a stable structure and the other weak C-H···O hydrogen bonds reinforced the packing (Fig. 2).

### Experimental

The title compound I was prepared by the literature method (Omar *et al.*, 2003). To a 100 mL flask provided with Dean–Stark tramp and magnetic stirrer was added (2-hydroxy-phenyl)-acetic acid (4.4 g, 29 mmol) in 60 mL of toluene and catalytic amounts of p-TsOH. The mixture was refluxed for 4 h with removal of water and then the residual solvent was removed at reduced pressure to give 3H-benzofuran-2-one in quantitative yield (3.9 g), mp 325K. Then, a mixture of concentrated nitric acid (4 ml) and glacial acetic acid (4 ml) was added drop wise to a solution of 3H-benzofuran-2-one (3.9 g) in acetic anhydride (25 ml) while the temperature was maintained below 293 K. The mixture was stirred and refluxed for 1 hour in ethanol (30 ml). The precipitate (the desired anthranilic acid esters ethyl 2-(2-hydroxy-5-nitrophenyl) acetate) was filtered off and washed with water, yield 80%. Crystals suitable for x-ray analysis were obtained by slow evaporation of an methanol solution.

### Refinement

H atoms were positioned geometrically, with O-H =1.92 Å (for OH) and C-H =0.93, 0.98 and 0.96 Å for aromatic, methine and methyl, respectively, and constrained to ride on their parent atoms, with U_iso_(H) = xU_eq_(C,N), where x = 1.5 for methyl H and x = 1.2 for all other H atoms.

### Crystal data

**Table:**

C_10_H_11_NO_5_	*F*(000) = 472
*M**_r_* = 225.20	*D*_x_ = 1.445 Mg m^−^^3^
Monoclinic, *P*2_1_/*c*	Melting point: 423 K
Hall symbol: -P 2ybc	Mo *K*α radiation, λ = 0.71073 Å
*a* = 11.066 (2) Å	Cell parameters from 25 reflections
*b* = 10.860 (2) Å	θ = 9–12°
*c* = 8.6970 (17) Å	µ = 0.12 mm^−^^1^
β = 97.85 (3)°	*T* = 293 K
*V* = 1035.4 (4) Å^3^	Block, yellow
*Z* = 4	0.30 × 0.20 × 0.10 mm

### Data collection

**Table:**

Enraf–Nonius CAD-4 diffractometer	1382 reflections with *I* > 2σ(*I*)
Radiation source: fine-focus sealed tube	*R*_int_ = 0.056
graphite	θ_max_ = 25.4°, θ_min_ = 1.9°
ω/2θ scans	*h* = 0→13
Absorption correction: ψ scan (North *et al.*, 1968)	*k* = −13→13
*T*_min_ = 0.966, *T*_max_ = 0.988	*l* = −10→10
3894 measured reflections	3 standard reflections every 200 reflections
1905 independent reflections	intensity decay: 1%

### Refinement

**Table:**

Refinement on *F*^2^	Primary atom site location: structure-invariant direct methods
Least-squares matrix: full	Secondary atom site location: difference Fourier map
*R*[*F*^2^ > 2σ(*F*^2^)] = 0.057	Hydrogen site location: inferred from neighbouring sites
*wR*(*F*^2^) = 0.153	H-atom parameters constrained
*S* = 1.00	*w* = 1/[σ^2^(*F*_o_^2^) + (0.099*P*)^2^] where *P* = (*F*_o_^2^ + 2*F*_c_^2^)/3
1905 reflections	(Δ/σ)_max_ < 0.001
145 parameters	Δρ_max_ = 0.59 e Å^−^^3^
0 restraints	Δρ_min_ = −0.19 e Å^−^^3^

### Special details

**Table:**

Geometry. All esds (except the esd in the dihedral angle between two l.s. planes) are estimated using the full covariance matrix. The cell esds are taken into account individually in the estimation of esds in distances, angles and torsion angles; correlations between esds in cell parameters are only used when they are defined by crystal symmetry. An approximate (isotropic) treatment of cell esds is used for estimating esds involving l.s. planes.
Refinement. Refinement of F^2^ against ALL reflections. The weighted R-factor wR and goodness of fit S are based on F^2^, conventional R-factors R are based on F, with F set to zero for negative F^2^. The threshold expression of F^2^ > 2sigma(F^2^) is used only for calculating R-factors(gt) etc. and is not relevant to the choice of reflections for refinement. R-factors based on F^2^ are statistically about twice as large as those based on F, and R- factors based on ALL data will be even larger.

### Fractional atomic coordinates and isotropic or equivalent isotropic displacement parameters (Å^2^)

**Table:**

	*x*	*y*	*z*	*U*_iso_*/*U*_eq_	
N	0.44757 (17)	0.77034 (17)	−0.2435 (2)	0.0459 (5)	
O1	0.84749 (14)	0.35380 (12)	0.03010 (16)	0.0410 (4)	
C1	0.9150 (3)	0.1519 (2)	−0.0185 (3)	0.0683 (9)	
H1A	0.9544	0.1015	−0.0874	0.102*	
H1B	0.8344	0.1209	−0.0133	0.102*	
H1C	0.9614	0.1503	0.0832	0.102*	
O2	0.86074 (14)	0.51755 (12)	−0.11869 (19)	0.0463 (4)	
C2	0.9070 (2)	0.2789 (2)	−0.0764 (3)	0.0514 (6)	
H2A	0.9880	0.3106	−0.0830	0.062*	
H2B	0.8604	0.2813	−0.1792	0.062*	
O3	0.83631 (14)	0.75835 (12)	0.23336 (18)	0.0447 (4)	
H3A	0.8435	0.8252	0.2774	0.067*	
C3	0.82574 (18)	0.46958 (16)	−0.0074 (2)	0.0328 (5)	
O4	0.40609 (16)	0.67242 (16)	−0.2982 (2)	0.0621 (5)	
C4	0.7546 (2)	0.53250 (18)	0.1045 (2)	0.0428 (6)	
H4A	0.6900	0.4778	0.1272	0.051*	
H4B	0.8082	0.5467	0.2008	0.051*	
C5	0.69929 (19)	0.65258 (17)	0.0481 (2)	0.0344 (5)	
O5	0.40459 (18)	0.86915 (17)	−0.2870 (2)	0.0748 (7)	
C6	0.60137 (19)	0.65579 (17)	−0.0694 (2)	0.0357 (5)	
H6A	0.5693	0.5828	−0.1137	0.043*	
C7	0.55121 (18)	0.76775 (18)	−0.1209 (2)	0.0360 (5)	
C8	0.5979 (2)	0.87793 (19)	−0.0587 (3)	0.0396 (5)	
H8A	0.5650	0.9525	−0.0968	0.048*	
C9	0.6931 (2)	0.87561 (18)	0.0595 (2)	0.0391 (5)	
H9A	0.7243	0.9491	0.1032	0.047*	
C10	0.74381 (18)	0.76365 (17)	0.1149 (2)	0.0332 (5)	

### Atomic displacement parameters (Å^2^)

**Table:**

	*U*^11^	*U*^22^	*U*^33^	*U*^12^	*U*^13^	*U*^23^
N	0.0430 (11)	0.0481 (11)	0.0477 (12)	0.0049 (9)	0.0104 (9)	0.0045 (9)
O1	0.0551 (10)	0.0265 (7)	0.0436 (9)	0.0072 (6)	0.0143 (7)	0.0031 (6)
C1	0.100 (2)	0.0364 (13)	0.0666 (18)	0.0172 (13)	0.0056 (16)	−0.0105 (12)
O2	0.0520 (9)	0.0380 (8)	0.0525 (10)	0.0080 (7)	0.0201 (7)	0.0112 (7)
C2	0.0547 (15)	0.0494 (13)	0.0546 (15)	0.0148 (11)	0.0232 (12)	−0.0012 (11)
O3	0.0476 (9)	0.0418 (9)	0.0440 (9)	0.0038 (7)	0.0040 (7)	−0.0097 (7)
C3	0.0372 (11)	0.0253 (9)	0.0353 (11)	0.0013 (8)	0.0026 (8)	0.0022 (8)
O4	0.0566 (11)	0.0569 (11)	0.0692 (12)	−0.0035 (9)	−0.0048 (9)	−0.0059 (9)
C4	0.0644 (15)	0.0314 (11)	0.0344 (11)	0.0096 (10)	0.0134 (10)	0.0023 (8)
C5	0.0467 (12)	0.0283 (10)	0.0318 (10)	0.0064 (8)	0.0181 (9)	−0.0006 (8)
O5	0.0697 (13)	0.0553 (11)	0.0918 (15)	0.0148 (9)	−0.0167 (11)	0.0160 (10)
C6	0.0454 (12)	0.0273 (10)	0.0371 (11)	0.0010 (8)	0.0162 (9)	−0.0039 (8)
C7	0.0378 (11)	0.0347 (10)	0.0381 (12)	0.0027 (8)	0.0149 (9)	−0.0021 (8)
C8	0.0487 (13)	0.0310 (10)	0.0424 (12)	0.0093 (9)	0.0178 (10)	0.0040 (9)
C9	0.0527 (13)	0.0268 (10)	0.0407 (12)	−0.0027 (9)	0.0169 (10)	−0.0037 (8)
C10	0.0372 (11)	0.0352 (10)	0.0307 (10)	0.0036 (8)	0.0171 (9)	−0.0032 (8)

### Geometric parameters (Å, °)

**Table:**

N—O5	1.213 (2)	C3—C4	1.498 (3)
N—O4	1.228 (2)	C4—C5	1.495 (3)
N—C7	1.455 (3)	C4—H4A	0.9700
O1—C3	1.313 (2)	C4—H4B	0.9700
O1—C2	1.456 (2)	C5—C6	1.385 (3)
C1—C2	1.466 (3)	C5—C10	1.399 (3)
C1—H1A	0.9600	C6—C7	1.385 (3)
C1—H1B	0.9600	C6—H6A	0.9300
C1—H1C	0.9600	C7—C8	1.384 (3)
O2—C3	1.208 (2)	C8—C9	1.369 (3)
C2—H2A	0.9700	C8—H8A	0.9300
C2—H2B	0.9700	C9—C10	1.397 (3)
O3—C10	1.351 (3)	C9—H9A	0.9300
O3—H3A	0.8200		
			
O5—N—O4	122.4 (2)	C3—C4—H4A	108.7
O5—N—C7	118.79 (18)	C5—C4—H4B	108.7
O4—N—C7	118.85 (17)	C3—C4—H4B	108.7
C3—O1—C2	117.42 (16)	H4A—C4—H4B	107.6
C2—C1—H1A	109.5	C6—C5—C10	118.72 (17)
C2—C1—H1B	109.5	C6—C5—C4	120.57 (18)
H1A—C1—H1B	109.5	C10—C5—C4	120.70 (19)
C2—C1—H1C	109.5	C5—C6—C7	119.96 (18)
H1A—C1—H1C	109.5	C5—C6—H6A	120.0
H1B—C1—H1C	109.5	C7—C6—H6A	120.0
O1—C2—C1	108.58 (19)	C8—C7—C6	121.4 (2)
O1—C2—H2A	110.0	C8—C7—N	118.96 (18)
C1—C2—H2A	110.0	C6—C7—N	119.64 (18)
O1—C2—H2B	110.0	C9—C8—C7	119.06 (18)
C1—C2—H2B	110.0	C9—C8—H8A	120.5
H2A—C2—H2B	108.4	C7—C8—H8A	120.5
C10—O3—H3A	109.5	C8—C9—C10	120.47 (18)
O2—C3—O1	122.96 (19)	C8—C9—H9A	119.8
O2—C3—C4	125.33 (17)	C10—C9—H9A	119.8
O1—C3—C4	111.71 (17)	O3—C10—C9	121.81 (18)
C5—C4—C3	114.36 (17)	O3—C10—C5	117.85 (17)
C5—C4—H4A	108.7	C9—C10—C5	120.34 (19)
			
C3—O1—C2—C1	−176.2 (2)	O4—N—C7—C8	−179.8 (2)
C2—O1—C3—O2	−5.6 (3)	O5—N—C7—C6	179.8 (2)
C2—O1—C3—C4	174.94 (19)	O4—N—C7—C6	0.5 (3)
O2—C3—C4—C5	15.6 (3)	C6—C7—C8—C9	−2.1 (3)
O1—C3—C4—C5	−164.98 (18)	N—C7—C8—C9	178.19 (18)
C3—C4—C5—C6	71.4 (3)	C7—C8—C9—C10	1.0 (3)
C3—C4—C5—C10	−109.9 (2)	C8—C9—C10—O3	−178.73 (19)
C10—C5—C6—C7	1.4 (3)	C8—C9—C10—C5	1.3 (3)
C4—C5—C6—C7	−179.79 (18)	C6—C5—C10—O3	177.52 (17)
C5—C6—C7—C8	0.9 (3)	C4—C5—C10—O3	−1.3 (3)
C5—C6—C7—N	−179.43 (17)	C6—C5—C10—C9	−2.5 (3)
O5—N—C7—C8	−0.4 (3)	C4—C5—C10—C9	178.75 (17)

### Hydrogen-bond geometry (Å, °)

**Table:**

*D*—H···*A*	*D*—H	H···*A*	*D*···*A*	*D*—H···*A*
O3—H3A···O2^i^	0.82	1.93	2.749 (2)	180
C2—H2A···O3^ii^	0.97	2.60	3.340 (3)	134
C4—H4A···O4^iii^	0.97	2.54	3.431 (3)	153
C6—H6A···O5^iv^	0.93	2.51	3.351 (3)	151
C8—H8A···O4^v^	0.93	2.59	3.430 (3)	150

Symmetry codes: (i) *x*, −*y*+3/2, *z*+1/2; (ii) −*x*+2, −*y*+1, −*z*; (iii) −*x*+1, −*y*+1, −*z*; (iv) −*x*+1, *y*−1/2, −*z*−1/2; (v) −*x*+1, *y*+1/2, −*z*−1/2.
